# Supplementary material for: The interactome of intact mitochondria by cross-linking mass spectrometry provides evidence for coexisting respiratory supercomplexes
Source: Mol Cell Proteomics. 2017 Dec 8;17(2):216–32. doi: 10.1074/mcp.RA117.000470 (PMC5795388; doi:10.1074/mcp.RA117.000470)
Supplement: Supplemental Data [file supp_RA117.000470_133922_0_supp_23978_fzffwf.zip › spectra_annotation/mito_DR_spectra_annotation/431-1-7-1-5-1.pdf]

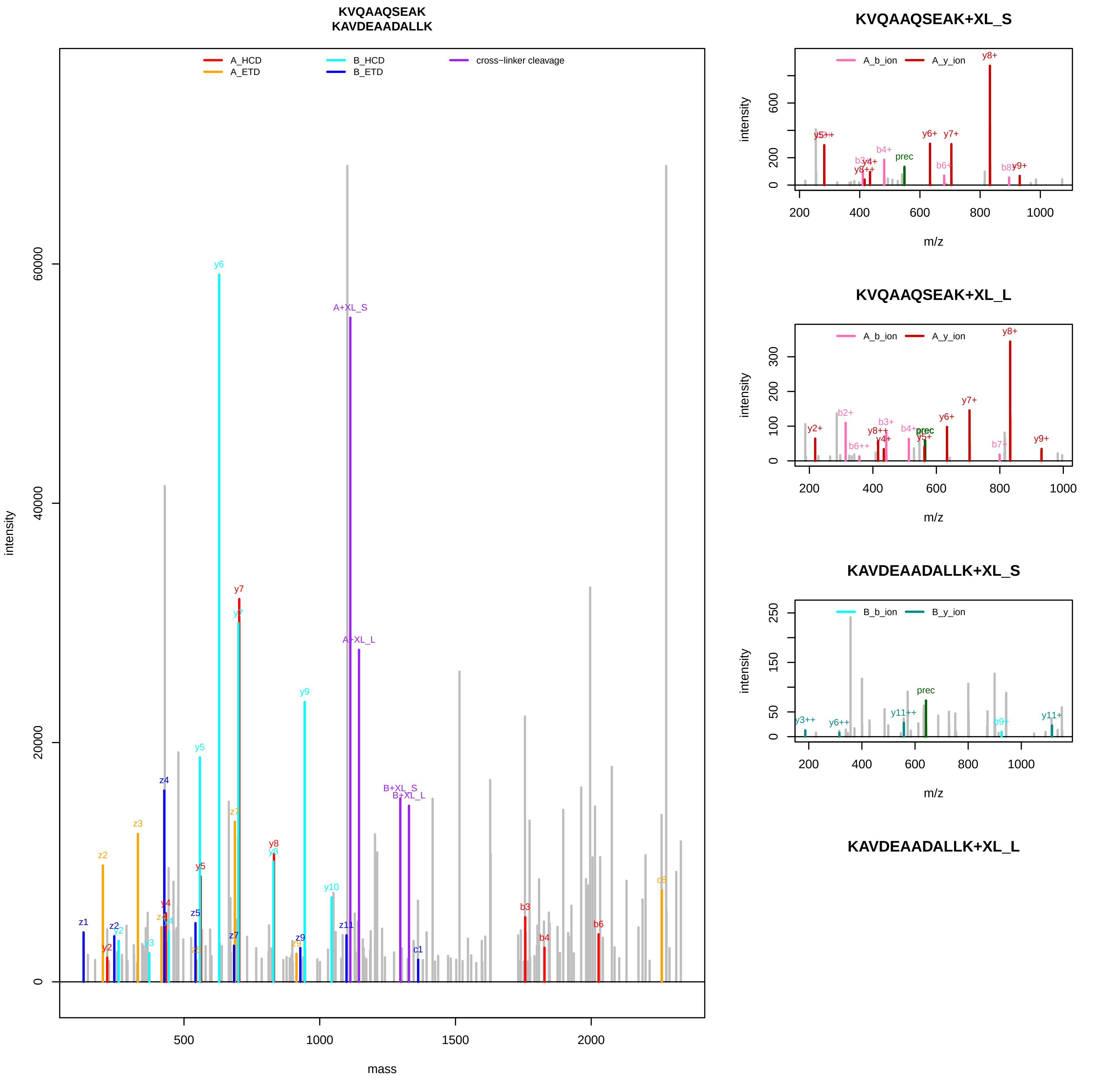
KVQAAQSEAK
KAVDEAADALLK
A_HCD
A_ETD
B_HCD
B_ETD
cross−linker cleavage
intensity
mass
0
20000
40000
60000
500
1000
1500
2000
y6
A+XL_S
A+XL_L
y7
y9
y5
z4
B+XL_S
B+XL_L
z7
z3
z2
y8
y5
y10
b3
b6
c8
z1
z5
z11
z9
b4
c1
KVQAAQSEAK+XL_S
A_b_ion
A_y_ion
m/z
y8+
y6+
y7+
b4+
prec
y9+
KVQAAQSEAK+XL_L
y2+
b2+
b3+
y8++
y4+
b4+
y5+
b6++
b7+
KAVDEAADALLK+XL_S
B_b_ion
B_y_ion
y3++
y6++
y11++
b9+
y11+
KAVDEAADALLK+XL_L
